# Correlation Between Spirituality and Quality of Life in People With Musculoskeletal Disorders: A Cross-Sectional Study

**DOI:** 10.7759/cureus.80055

**Published:** 2025-03-04

**Authors:** Tushara Nair, G. Palani Kumar

**Affiliations:** 1 College of Physiotherapy, Sumandeep Vidyapeeth (Deemed to Be University), Vadodara, IND

**Keywords:** daily spiritual experience scale, musculoskeletal disorders, pain, quality of life, spirituality

## Abstract

Background

The prevalence of musculoskeletal disorders (MSKDs) is increasing day by day in industrially growing countries like India. Spirituality forms the roots of Indian culture, and according to the World Health Organization (WHO), it is one of the important factors affecting health. Many studies have concluded the correlation between spirituality and quality of life (QoL), but when works of literature were searched on PubMed, Scopus, and Web of Science, no such study was conducted in India on people with MSKDs. Hence, the aim of this study is to find out the correlation between spirituality and QoL in patients with MSKDs.

Methods

In this cross-sectional study, 161 patients with MSKDs coming to Dhiraj General Hospital, Vadodara, India, from October 2023 to December 2023, fulfilling the inclusion and exclusion criteria, were included. The demographic data and the site of pain were recorded. The severity of pain was assessed using the Numerical Pain Rating Scale (NPRS). The Daily Spiritual Experience Scale (DSES) and RAND 36-Item Health Survey (Version 1.0) were used to assess spirituality and QoL.

Results

DSES was found to have a fair correlation with NPRS with a p-value of 0.013. Among the eight components of RAND-36, energy, emotional well-being, social function, pain, and general health were found to have a positive correlation with DSES with p-values of 0.008, 0.001, 0.009, 0.032, and <0.001, respectively, whereas no correlation was found between DSES and other components of RAND-36.

Conclusion

This study provides empirical data about how spirituality is positively related to different aspects of QoL. This can be further useful in planning integrated healthcare approaches based on spirituality for better QoL in patients with MSKDs.

## Introduction

Musculoskeletal disorders (MSKDs) are becoming one of the major issues due to the increase in their prevalence in developing countries like India [1,2]. The quality of life (QoL) of people with MSKDs like low back pain, knee pain, neck pain, etc. gets affected as they have decreased mobility, an increase in pain, and additional hospital expenses, which increase their dependency on others [3].

Spirituality forms the roots of Indian culture and has been identified as an important factor affecting QoL and health by the World Health Organization (WHO) [4,5]. Spirituality is basically defined as “the way people connect to their own self, to the present, to others, to nature and to the divine” [6]. According to Indian philosophy, spirituality is the union of individual consciousness (own self) with the universal consciousness (divine) [4]. A spiritual person learns to manage a stressful event by constantly adapting his behavior and thinking toward such negative events, which is termed “spiritual coping.” This improves bodily, mental, and social well-being, which helps in self-realization [7,8]. Spiritual coping also inculcates hope in patients, which helps the patients to deal with their bodily aches and pains in a better way. Patients who are highly spiritual use tactics such as finding logical solutions to problems, moving away from negativity, positive feedback, self-discipline, and accepting help from others, which aids in improving their QoL [9,10].

Many studies have concluded the correlation between spirituality and QoL, but to the best of our knowledge, no such study is available on PubMed, Scopus, or Web of Science that has been conducted in India on people with MSKDs. Hence, the aim of this study is to find out the correlation between spirituality and QoL in patients with MSKDs.

This article was previously presented as a poster at the Ninth International Conference of Physical Therapy (INCPT) All India Institute of Medical Sciences (AIIMS) 2023 on December 23 and 24, 2023.

## Materials and methods

This study was approved by the Sumandeep Vidyapeeth Institutional Ethics Committee, Sumandeep Vidyapeeth (Deemed to Be University), Vadodara, India, on October 16, 2023, with the approval number SVIEC/ON/Phys/RP/OC/123/4. The above study has also been registered in the Clinical Trials Registry India (CTRI), and the CTRI number is CTRI/2024/03/064123.

Study design

This cross-sectional study was conducted for a time-bound period of three months from October 2023 to December 2023 at the Outdoor Physiotherapy Department (OPD) of Dhiraj General Hospital (DGH), Vadodara, India. The sample size was calculated with the following formula:

$n = \left( \frac{Z_{\alpha/2}^2 \cdot P(1 - P)}{d^2} \right)$

where prevalence (P) was found to be 42% and an allowable error (d) of 8%, and due to non-response the obtained sample size was increased by 10%. Hence, the calculated sample size was found to be 161, and the method of sampling was convenient. Patients diagnosed with any MSKD by a physiotherapist or an orthopedic surgeon were included in the study based on the inclusion and exclusion criteria.

Inclusion criteria

Patients aged 18 or older, diagnosed with any MSKD for at least three months, and those who can read and understand Hindi, Gujarati, or English, were included in the study.

Exclusion criteria

Patients with any major musculoskeletal, traumatic, or congenital orthopedic conditions; cardiopulmonary, neurological, psychosomatic, or cognitive impairments; or a recent history of any surgery within three months were excluded.

Procedure

All the subjects diagnosed with any MSKD by an orthopedic surgeon or physiotherapist willing to participate in the study were included based on the inclusion and exclusion criteria. The subjects were explained about the study and asked to sign the consent form. A thorough physiotherapy examination was done, and the following outcome measures were taken: The Daily Spiritual Experience Scale (DSES) and RAND-36 Version 1.0.

Variables and instruments

The DSES was used to assess spirituality. The permission to use and translate this scale was taken from the original author. It is a 16-item self-administered questionnaire developed for use in healthcare, and it assesses the daily spiritual experiences of an individual [11]. The items in the questionnaire referred to both spirituality and religiosity. Most of the items in DSES were theistic, but some of the items in it were also designed to measure the spiritual experiences of those who had an atheistic view. This scale has been used in over 70 published studies [12]. This scale is originally available in English and Hindi. Considering cross-cultural adaptation, this scale was translated into Gujarati by language experts, and face validation was conducted by 10 experts.

The QoL was assessed using the RAND 36-Item Health Survey (Version 1.0), which consists of eight components: physical function, pain, role limitation due to physical health problems, role limitation due to emotional problems, emotional well-being, social functioning, energy/fatigue, and general health. This scale is available in English and Gujarati and is found to have good reliability and validity [13,14]. Due to the unavailability of RAND-36 in Hindi, it was translated into Hindi by language experts, and face validation was done by 10 experts.

IBM SPSS Statistics for Windows, Version 20 (Released 2011; IBM Corp., Armonk, New York, United States) was used for the statistical analysis. The baseline data was analyzed using the Kolmogorov-Smirnov test, and the correlation was checked using Spearman’s rank correlation coefficient.

## Results

**Figure 1:**
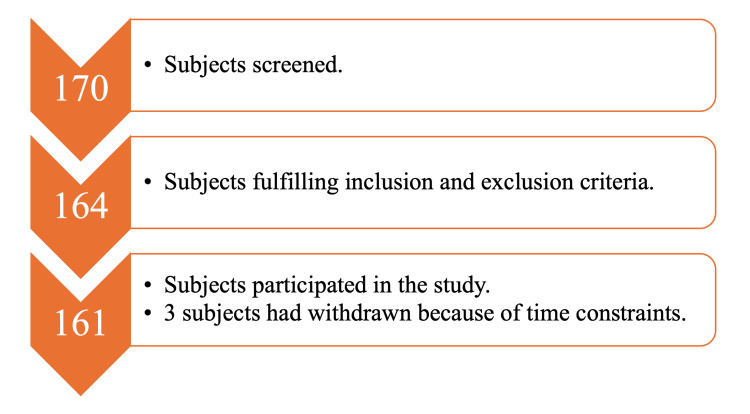
Flow diagram representing data collection

A total of 170 patients diagnosed with any MSKD by an orthopedic surgeon or physiotherapist referred to the OPD of DGH, Vadodara, India, were screened. Out of these, 161 patients willing to participate were included based on the inclusion and exclusion criteria. Three patients had withdrawn because of time constraints. The flow diagram representing the data collection is shown in Figure 1.

The normality was checked using the Kolmogorov-Smirnov test for the baseline data, age, and the p-value at a 95% confidence interval is depicted in Table 1. There was no statistically significant difference in the baseline data of the subjects, and the data followed the normal distribution curve.

**Table 1:** Baseline characteristics SD: standard deviation; p: probability; D: distance

Variable	Mean±SD	p-value	Kolmogorov-Smirnov statistic (D)
Age	40.73±15.58	0.01845	0.1195

The demographic data regarding the percentage distribution of females:males and the joints affected is shown in Table 2.

**Table 2:** Demographic data

Demographic data	Variable	Percentage (%)
Gender distribution	Female	64
	Male	36
Joint affection	Lower back	43
	Knee	20
	Upper back	12
	Neck	11
	Shoulder	7
	Ankle	2
	Feet	2
	Wrist	1
	Hip	1
	Elbow	1

The correlation between DSES and different components of RAND-36 and the Numerical Pain Rating Scale (NPRS) was calculated using Spearman’s rank correlation coefficient, and the p-value at a 95% confidence interval is given in Table 3.

**Table 3:** Correlation between DSES, NPRS, and QoL DSES: Daily Spiritual Experience Scale; NPRS: Numerical Pain Rating Scale; p: probability; QoL: quality of life

Variable	Correlation with DSES	
	R (rho)	p-value
RAND-36 physical function	0.025	0.757
RAND-36 role limitation due to physical health	-0.099	0.212
RAND-36 role limitation due to emotional problem	-0.131	0.096
RAND-36 energy	-0.208	0.008
RAND-36 emotional well-being	-0.248	0.001
RAND-36 social function	-0.206	0.009
RAND-36 pain	-0.168	0.033
RAND-36 general health	-0.272	<0.001
NPRS	-0.196	0.013

The lower value of DSES indicates higher spirituality and higher values of RAND-36 indicate better QoL, hence negative correlation was found between DSES and five components of RAND-36, that is energy, emotional well-being, social function, pain, and general health with a p-value of less than 0.05, whereas no correlation was found with physical function, role limitation due to physical health and emotional problems. A negative correlation was found between DSES and NPRS with a p-value of less than 0.05.

**Figure 2:**
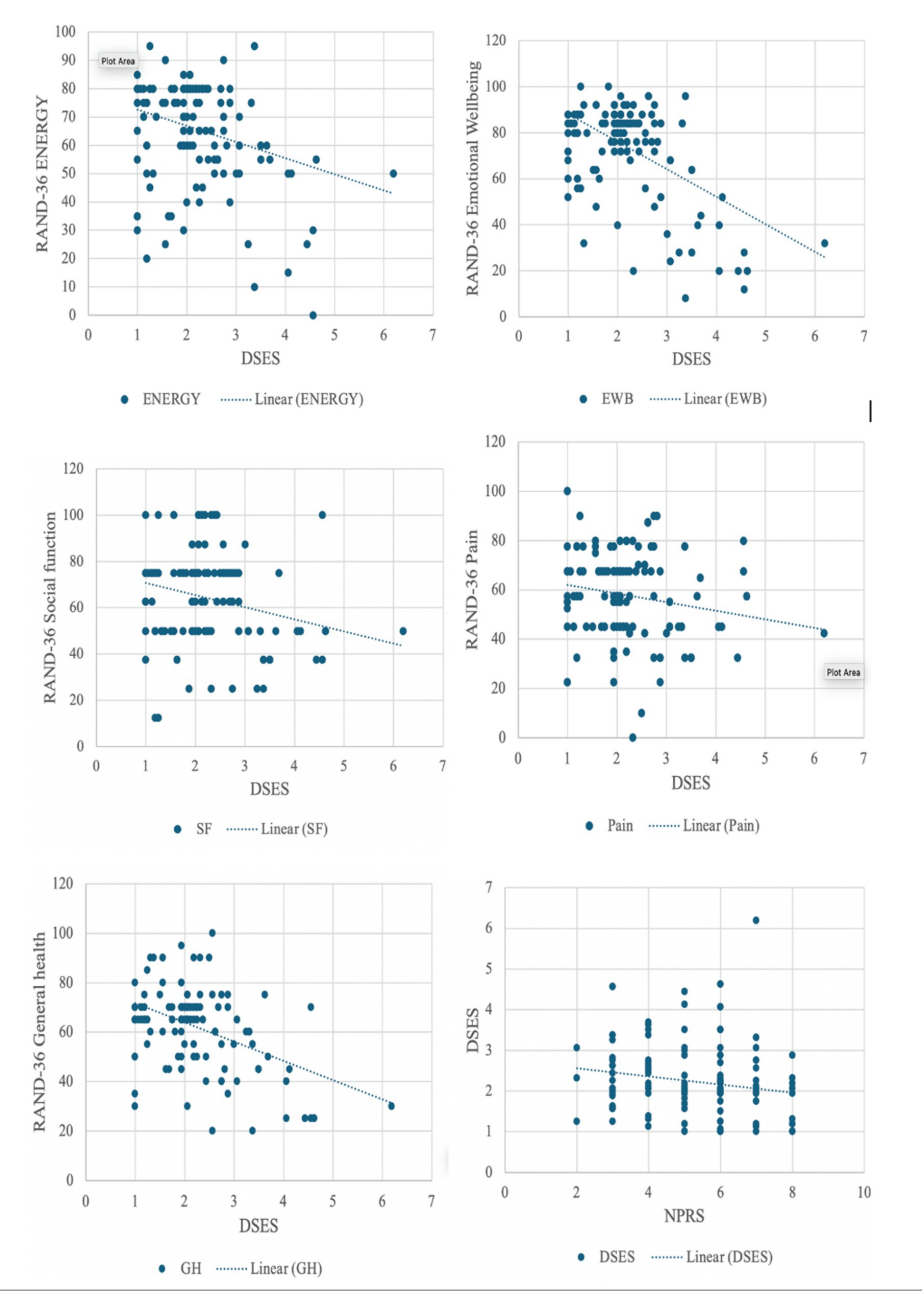
Correlation of DSES with different components of RAND-36 and NPRS DSES: Daily Spiritual Experience Scale; NPRS: Numerical Pain Rating Scale; EWB: emotional well-being; SF: social function; GH: general health

The scatter diagram indicating the negative correlation between DSES and five components of RAND-36, that is energy, emotional well-being, social function, pain, and general health and NPRS is depicted in Figure 2. This describes that as the spirituality increases, the QoL improves and as the value of DSES decreases, the value of NPRS increases.

## Discussion

In the present study, the correlation between spirituality and QoL was studied. Spirituality was assessed using DSES. Underwood, in the year 2011, developed and studied the psychometric properties of this scale and concluded that this scale had good reliability and validity [12]. This scale has also been used in more than 70 published studies [11]. Hence, DSES was used to assess spirituality.

RAND-36 is one of the commonly used scales to assess QoL. VanderZee et al. studied the psychometric properties of the RAND-36-Item Health Survey 1.0 on 1063 participants in the year 1996. This study concluded that RAND-36 had good reliability and validity [13]. Hence, it was used to assess the QoL.

The results concluded that a negative correlation was found between DSES and five components of RAND-36, that is, energy, emotional well-being, social function, pain, and general health. The lower the score of DSES, the higher the level of spirituality; because of this, the emotional aspects and general health improve, which increases the score of RAND-36. Therefore, the correlation value was found to be negative.

Bai et al. studied the relationship between spirituality and QoL in 102 Black patients with cancer pain and concluded that spirituality is associated with decreased pain and improves the overall QoL, specifically social, emotional, and functional domains, which supports our study [15].

No correlation was found between DSES and other components of RAND-36, that is, physical function, role limitation due to physical health, and emotional problems.

Aloush et al. studied the relationship between religiosity, spirituality, and physical and mental outcomes in 55 patients with fibromyalgia, where they concluded that spirituality was negatively correlated with role limitation due to physical health and emotional problems, which supports our study [16].

A negative correlation was found between DSES and NPRS. Moreira-Almeida and Koenig in the year 2006 studied the relationship of religiousness and spirituality in chronic pain patients and concluded that religious variables are not usually associated with pain variables, which supports our study [17].

Most elderly people in India are inclined toward spirituality, and due to the physiological aging process, their pain levels were found to be high. This can also be one of the reasons for the negative correlation.

The present study concludes that spirituality, which is the independent variable, was found to have a positive correlation with the dependent variable, which is QoL. However, the results derived from this study are difficult to generalize to the whole population, as the data were collected from a single hospital, and the method of sampling was convenient. Other confounding factors affecting the QoL, like occupation, socio-economic status, working hours, etc., were not taken into consideration. Furthermore, only the daily spiritual experiences were measured, which included the overlapping parts of spirituality and religiosity; both parts were not assessed differently.

## Conclusions

MSKDs are one of the most common conditions prevalent in all occupations and are also becoming a major economic concern for developing countries like India. Spirituality forms the roots of Indian culture, and it has been found that spirituality highly influences the QoL of patients with MSKDs in a positive way. The current study also concluded that spirituality had a positive correlation with different components of QoL. This data can be further used to plan integrated health care approaches, including spirituality, to improve the QoL of patients with MSKDs. Hence, studies like this can prove to be beneficial in decreasing the disease burden and the economic losses incurred as a result of the disease.

## Appendices

Appendix A

**Table 4:** Daily Spiritual Experience Scale (DSES) - English version Source: The original author Lynn G. Underwood was approached and the English questionnaire was obtained by mail. Permission to reproduce this questionnaire has been obtained. Questionnaire: The list that follows includes items you may or may not experience. Please consider how often you directly have this experience, and try to disregard whether you feel you should or should not have these experiences. A number of items use the word "God." If this word is not a comfortable one for you, please substitute another word that calls to mind the divine or holy for you. Put a tick in front of the option which seems appropriate to you in the following sentences.

		Many times a day	Every day		Most days	Some days	Once in a while		Never or almost never
1	I feel God’s presence.								
2	I experience a connection to all of life.								
3	During worship, or at other times when connecting with God, I feel joy which lifts me out of my daily concerns.								
4	I find strength in my religion or spirituality.								
5	I find comfort in my religion or spirituality.								
6	I feel deep inner peace or harmony.								
7	I ask for God’s help in the midst of daily activities.								
8	I feel guided by God in the midst of daily activities.								
9	I feel God’s love for me directly.								
10	I feel God’s love for me through others								
11	I am spiritually touched by the beauty of creation.								
12	I feel thankful for my blessings.								
13	I feel a selfless caring for others.								
14	I accept others even when they do things I think are wrong.								
15	I desire to be closer to God or in union with the divine.								
		Not close		Somewhat close		Very close		As close as possible	
16	In general, how close do you feel to God?								

Appendix B

**Table 5:** Daily Spiritual Experience Scale (DSES) - Hindi Translation by Vibhuti Gupta and Lynn G. Underwood Draft Hindi translation February 2012 © Lynn G. Underwood www.dsescale.org Do not copy without permission of the author. Reference: [11] Source: The original author Lynn G. Underwood was approached and the Hindi questionnaire was obtained. Permission to reproduce this questionnaire has been obtained. Questionnaire: नीचे दी गई सूची (list) में ऐसी बातें शामिल हैं जिनका आप अनुभव करते हों या न करते हों।  कृपया विचार कीजिये की आप प्रायः कितनी बारऐसा सीधा अनुभव करते हैं तथा इस पर ध्यान न दें की आपको यह अनुभव होना चाहिए या नहीं।  कई बातों में "ईश्वर" शब्द का उपयोग कियागया हैं। यदि यह शब्द आप क लिए सुविधाजनक नहीं है तो इसका विकल्प रखें जोह आप के मन में दिव्यता या पवित्रता लाता हो। निम्नलिखित वाक्यों में से जो विकल्प आपको उचित लगे उस के आगे (✓) का प्रयोग करें।

		दिन मेंकई बार	हर रोज	ज्यादातरदिन	कुछदिन		कभीकभार	कभी नहीं
1	मैं ईश्वर की उपस्थिति महसूस करता/ करती हूँ।							
2	मैं अनुभव करता/ करती हूँ कि मैं सभी जीवन से जुड़ा / जुडी हूँ।							
3	प्रार्थना के दौरान या अन्य समय में ईश्वर से जुड़ते हुए मैं आनंद अनुभव करता / करती हूँ जोह मुझे मेरी दैनिक चिंताओं से बहार निकलता है।							
4	मैं अपने धर्म या अध्यात्म में शक्ति पाता/ पाती हूँ।							
5	मैं अपने धर्म या अध्यात्म में आराम महसूस करता/ करती हूँ।							
6	मुझे आंतरिक शांति या सुकून महसूसहोता है।							
7	मैं दैनिक कार्यों के बीच ईश्वर से सहायता मांगता/ मांगती हूँ।							
8	मैं दैनिक कार्यों के बीच ईश्वर का मार्गदर्शन हुआ महसूस होता है।							
9	मुझे ईश्वर के प्रेम की सीढ़ी अनुभूति होती है।							
10	मैं अन्य लोगों के माध्यम से अपने प्रतिईश्वर का प्रेम को महसूस करता हूँ।							
11	सृष्टि की सुंदरता ने मुझे आध्यात्मिक रूप से स्पर्श किया है।							
12	मुझे मिले आशीर्वादों के लिए मैं आभारमहसूस करता/ करती हूँ।							
13	मुझे अन्य लोगों के प्रति निःस्वार्थदेखभाल की भावना महसूस करता/ करती हूँ।							
14	मैं औरों को तब भी स्वीकार करता/ करती हूँ, जब वे मेरे विचार से गलत कार्य करते है।							
15	मेरी इच्छा है कि मैं ईश्वर के और निकट आ जाऊँ या दिव्यता में एकाकार हो जाऊँ।							
		बिलकुल नहीं	कुछ निकट	बहुत निकट		यथासंभव निकट (जितना हो सके उतना)		
16	सामान्य रूप में, आप ईश्वर के कितना निकट महसूस करते हैं?							

Appendix C

**Table 6:** Daily Spiritual Experience Scale (DSES) - Gujarati Source: The original author Lynn G. Underwood was approached and permission was taken to translate the scale into Gujarati. The original author Lynn G. Underwood also took part in the back translation stage. Permission to reproduce this questionnaire has been obtained. Questionnaire: નીચે આપેલી સૂચિમાં તે બાબતોનો સમાવેશ કરેલો છે જે તમે અનુભવી શકો અથવા ન અનુભવી શકો. કૃપા કરીને વિચારો તમને કેટલી વાર સીધો આ અનુભવ થાય છે, અને આવા અનુભવો હોવા જોઈએ કે ન હોવા જોઈએ એને ધ્યાનમાં ના લેતા. ઘણી બાબતોમાં ‘ઈશ્વર’ શબ્દનો ઉપયોગ કરવામાં આવેલો છે. જો આ શબ્દ તમારા માટે સંમત નથી, તો આ શબ્ધ ની જગ્યાએ જે તમારા મનમાં દિવ્યતા અથવા પવિત્રતા લાવતા હોય એનો પ્રયોગ કરી શકો છો. નીચેના વાક્યોમાં તમને યોગ્ય લાગે તેવા વિકલ્પની સામે ટિક કરો.

		દિવસમાં ઘણી વખત	દરરોજ		અધિકતર દિવસ		અમુક દિવસ	ક્યારેક		ક્યારેય નહિ અથવા લગભગ ક્યારેય નહિ
1	હું ઈશ્વરની હાજરી અનુભવું છું.									
2	હું બધા જીવન સાથે જોડાણ અનુભવું છું.									
3	પૂજા દરમિયાન, અથવા અન્ય સમયે જ્યારે ઈશ્વર સાથે જોડાવું ત્યારે હું આનંદ અનુભવું છું જે મને મારી દૈનિક ચિંતાઓમાંથી બહાર કાઢે છે.									
4	મને મારા ધર્મ અથવા આધ્યાત્મિકતા માં તાકાત મળે છે.									
5	મને મારા ધર્મ અથવા આધ્યાત્મિકતા માંઆશ્વાસન મળે છે.									
6	હું ગહન આંતરિક શાંતિ અથવા સંવાદિતા અનુભવું છું.									
7	હું દૈનિક પ્રવૃત્તિઓ વચ્ચે ઈશ્વરથી મદદ માંગુ છું.									
8	હું દૈનિક પ્રવૃત્તિઓ વચ્ચેઈશ્વર દ્વારા માર્ગદર્શન અનુભવું છું.									
9	હું મારા માટે ઈશ્વરનો પ્રેમઅનુભવું છું.									
10	હું અન્ય લોકો દ્વારા મારા માટે ઈશ્વરનો પ્રેમ અનુભવું છું.									
11	હું સર્જન ની સુંદરતાથી આધ્યાત્મિક રીતે પ્રભાવિત છું.									
12	હું મને મળેલા આશીર્વાદોમાટે આભાર માનું છું.									
13	હું અન્ય લોકો માટે નિઃસ્વાર્થ કાળજી અનુભવું છું.									
14	હું બીજાને ત્યારે પણ સ્વીકારું છું જ્યારે તેઓ મને ખોટું લાગે એવું કામ કરે.									
15	હું ઈશ્વરની નજીક અથવાપરમાત્મા સાથે જોડાવા ઈચ્છું છું.									
		નજીક નથી		લગભગ નજીક		એકદમ નજીક			જેટલું નજીક શક્ય હોય	
16	સામાન્ય રીતે, તમે ઈશ્વરની કેટલી નજીક અનુભવો છો?									

Appendix D

RAND 36-Item Health Survey 1.0 Questionnaire Items (English Version)

Source: The scale was downloaded from the original website https://www.rand.org/content/dam/rand/www/external/health/surveys_tools/mos/mos_core_36item_survey.pdf

Permission to reproduce this questionnaire has been obtained.

Questionnaire:

Choose one option for each questionnaire item. 

1. In general, would you say your health is: 

◯ 1 - Excellent 

◯ 2 - Very good 

◯ 3 - Good

◯ 4 - Fair

◯ 5 - Poor

2. Compared to one year ago, how would you rate your health in general now?  

◯ 1 - Much better now than one year ago

◯ 2 - Somewhat better now than one year ago

◯ 3 - About the same 

◯ 4 - Somewhat worse now than one year ago  

◯ 5 - Much worse now than one year ago  

The following items are about activities you might do during a typical day. Does your health now limit you in these activities? If so, how much? (Table 7)

**Table 7:** RAND 36 - Item Health Survey 1.0 Questionnaire items

		Yes, limited a lot 1	Yes, limited a little 2	No, not limited at all 3
3	Vigorous activities, such as running, lifting heavy objects, participating in strenuous sports			
4	Moderate activities, such as moving a table, pushing a vacuum cleaner, bowling, or playing golf			
5	Lifting or carrying groceries			
6	Climbing several flights of stairs			
7	Climbing one flight of stairs			
8	Bending, kneeling, or stooping			
9	Walking more than a mile			
10	Walking several blocks			
11	Walking one block			
12	Bathing or dressing yourself			

During the past 4 weeks, have you had any of the following problems with your work or other regular daily activities as a result of your physical health? (Table 8)

**Table 8:** RAND 36 - Item Health Survey 1.0 Questionnaire items

		Yes 1	No 2
13	Cut down the amount of time you spent on work or other activities		
14	Accomplished less than you would like		
15	Were limited in the kind of work or other activities		
16	Had difficulty performing the work or other activities (for example, it took extra effort)		

During the past 4 weeks, have you had any of the following problems with your work or other regular daily activities as a result of any emotional problems (such as feeling depressed or anxious)? (Table 9)

**Table 9:** RAND 36 - Item Health Survey 1.0 Questionnaire items

		Yes 1	No 2
17	Cut down the amount of time you spent on work or other activities		
18	Accomplished less than you would like		
19	Didn't do work or other activities as carefully as usual		

20. During the past 4 weeks, to what extent has your physical health or emotional problems interfered with your normal social activities with family, friends, neighbors, or groups? 

◯ 1 - Not at all

◯ 2 - Slightly

◯ 3 - Moderately 

◯ 4 - Quite a bit 

◯ 5 - Extremely 

21. How much bodily pain have you had during the past 4 weeks?

◯ 1 - None 

◯ 2 - Very mild 

◯ 3 - Mild

◯ 4 - Moderate 

◯ 5 - Severe 

◯ 6 - Very severe 

22. During the past 4 weeks, how much did pain interfere with your normal work (including both work outside the home and housework)? 

◯ 1 - Not at all

◯ 2 - A little bit 

◯ 3 - Moderately 

◯ 4 - Quite a bit 

◯ 5 - Extremely

These questions are about how you feel and how things have been with you during the past 4 weeks. For each question, please give the one answer that comes closest to the way you have been feeling. 

How much of the time during the past 4 weeks... (Table 10)

**Table 10:** RAND 36 - Item Health Survey 1.0 Questionnaire items

		All of the time 1	Most of the time 2	A good bit of time 3	Some of the time 4	A little of the time 5	None of the time 6
23	Did you feel full of pep?						
24	Have you been a very nervous person?						
25	Have you felt so down in the dumps that nothing could cheer you up?						
26	Have you felt calm and peaceful?						
27	Did you have a lot of energy?						
28	Have you felt downhearted and blue?						
29	Did you feel worn out?						
30	Have you been a happy person?						
31	Did you feel tired?						

32. During the past 4 weeks, how much of the time has your physical health or emotional problems interfered with your social activities (like visiting with friends, relatives, etc.)? 

◯ 1 - All of the time

◯ 2 - Most of the time

◯ 3 - Some of the time 

◯ 4 - A little of the time 

◯ 5 - None of the time 

How TRUE or FALSE is each of the following statements for you.

**Table 11:** RAND 36 - Item Health Survey 1.0 Questionnaire items

		Definitely true 1	Mostly true 2	Don’t know 3	Mostly false 4	Definitely false 5
33	Did you feel full of pep?					
34	Have you been a very nervous person?					
35	Have you felt so down in the dumps that nothing could cheer you up?					
36	Have you felt calm and peaceful?					

Appendix E

RAND 36-Item Health Survey 1.0 Questionnaire Items (Hindi Version)

Source: This original scale which was available in English was translated into Hindi by language experts and expert opinion was taken from 10 experts.

Permission to reproduce this questionnaire has been obtained.

Questionnaire: (Tables 12-17)

**Table 12:** RAND 36 - Item Health Survey (Hindi)

१. सामान्य तौर पर, क्या आप कहेंगे कि आपका स्वास्थ्य ऐसा है:	
उत्तम	१
बहुत अच्छा	२
अच्छा	३
ठीक ठाक	४
कमज़ोर	५
२. एक साल पहले की तुलना में अब आप अपने स्वास्थय को सामान्य रूप से कैसेमूल्यांकन करेंगे?	
एक साल पहले की तुलना में अब काफी बेहतर है	१
एक साल पहले की तुलना में अब कुछ बेहतर है	२
लगभग एक ही	३
एक साल पहले की तुलना में अब स्थिति कुछ हद तक खराब है	४
एक साल पहले की तुलना में अब काफी खराब स्थिति है	५

निम्नलिखित आइटम उन प्रवृत्तियों के बारे में हैं जो आप एक सामान्य दिन के दौरान कर सकते हैं। क्या आपका स्वास्थ्य अब आपको इन प्रवृत्तियों मेंसीमित करता है? यदि हां, तो कितना?

(प्रत्येक पंक्ति पर एक अंक का गोला बनाएं) 

**Table 13:** RAND 36 - Item Health Survey (Hindi)

	हाँ, बहुत मर्यादित	हां, थोड़ा मर्यादित	ना, बिलकुलमरयित नहीं
३. जोशीले प्रवृत्तियाँ, जैसे दौड़ना, भारी वस्तुएं उठाना, कठिन खेल-कूद में भाग लेना	१	२	३
४. मध्यम प्रवृत्तियाँ, जैसे मेज़ हिलाना, वैक्यूम क्लीनरको धकेलना, गेंदबाजी करना, या गोल्फ खेलना	१	२	३
५. किराने का सामान उठाना या ले जाना	१	२	३
६. कई सीढ़ियाँ चढ़ना	१	२	३
७. सीढि़यों का एक समूह चढ़ना	१	२	३
८. झुकना, घुटने टेकना या घुटना मोड़ के झुकना	१	२	३
९. एक मील से ज्यादा चलना	१	२	३
१०. कई ब्लॉक चलना	१	२	३
११. एक ब्लॉक चलना	१	२	३
१२. नहाना या स्वयं कपड़े पहनना	१	२	३

पिछले ४ सप्ताहों के दौरान, क्या आपको अपने काम या अन्य नियमित दैनिक कार्यों में आपके शारीरिक स्वास्थ्य के परिणाम स्वरूप निम्नलिखित में सेकोई समस्या हुई है?

(प्रत्येक पंक्ति पर एक अंक का गोला बनाएं)

**Table 14:** RAND 36 - Item Health Survey (Hindi)

	हा	ना
१३. आपने काम या अन्य प्रवृत्तियों पर खर्च होने वाले समय में कटौती किया है	१	२
१४. आपने जितना चाहा उससे कम पूरा हुआ	१	२
१५. काम या अन्य प्रकार के प्रवृत्तियों में सीमित हे	१	२
१६. कार्य या अन्य प्रवृत्तियाँ करने में कठिनाई होती है (उदाहरण के लिए, अतिरिक्त प्रयासकरना)	१	२

पिछले ४ सप्ताहों के दौरान, क्या आपको अपने काम या अन्य नियमित दैनिक कार्यों में अपने भावनात्मक समस्याओ (जैसे उदास या चिंतित महसूसकरना) के कारण  कोई समस्या हुई है? 

(प्रत्येक पंक्ति पर एक अंक का गोला बनाएं)

**Table 15:** RAND 36 - Item Health Survey (Hindi)

	हा	ना
१७. काम या अन्य प्रवृत्तियों पर खर्च होने वाले समय में कटौती करते है	१	२
१८. आपने जितना चाहा उससे कम पूरा हुआ	१	२
१९. काम या अन्य प्रवृत्तियाँ हमेशा की तरह सावधानी से नहीं की	१	२

२०. पिछले ४ हफ्तों के दौरान आपके शारीरिक स्वास्थ्य या भावनात्मक समस्याओं ने किस हद तक आपकी सामान्य सामाजिक परिवार, दोस्तों, पड़ोसियों या समूहों के साथ के प्रवृत्तियों में हस्तक्षेप किया है ?

           (एक नंबर पर गोला लगाएं)

            बिल्कुल नहीं १

            थोड़ा २

            मध्यम ३

            बहुत थोड़ा ४

            अत्यंत ५

२१. पिछले ४ सप्ताह के दौरान आपको कितना शारीरिक दर्द हुआ है?

           (एक नंबर पर गोला लगाएं)

बिल्कुल नहीं १

बहुत हल्का २

हल्का ३

मध्यम ४

गंभीर ५

बहुत गंभीर ६

२२. पिछले ४ सप्ताहों के दौरान, दर्द ने आपके सामान्य कार्य (घर से बाहर और घर का काम दोनों कार्यों सहित) में कितना हस्तक्षेप किया ?

            (एक नंबर पर गोला लगाएं)

            बिल्कुल नहीं १

            थोड़ा २

            मध्यम ३

            बहुत थोड़ा ४

            अत्यंत ५

ये प्रश्न इस बारे में हैं कि पिछले ४ सप्ताह के दौरान आप कैसा महसूस करते हैं और चीजें आपके साथ कैसी रही हैं। प्रत्येक प्रश्न के लिए कृपया वहीउत्तर दें जो आपके अनुभव के सबसे निकट हो।

पिछले ४ सप्ताह के दौरान कितना समय. . .

(प्रत्येक पंक्ति पर एक अंक का गोला बनाएं)

**Table 16:** RAND 36 - Item Health Survey (Hindi)

	सभी समय	ज्यादातरसमय	अच्छा ख़ासासमय	कुछ समय	थोड़ा समय	कोई समयनहीं
२३. क्या आप उत्साह से भरा हुआ महसूसकरते हो?						
२४. क्या आप एक घबराये हुए व्यक्ति है ?						
२५. क्या आप इतना उदास महसूस करते हैके आपको कुछ भी उत्साहित न करसके?						
२६. क्या आपने शांति महसूस की है?						
२७. क्या आपके पास बहुत ऊर्जा है?						
२८. क्या आपने निराशा या हताशा महसूसकी है?						
२९. क्या आप थका हुआ महसूस करते है?						
३०. क्या आप एक खुश इंसान है?						
३१. क्या आपको थकान महसूस हुई?						

३२. पिछले ४ हफ्तों के दौरान, कितने समय आपके शारीरिक स्वास्थ्य या भावनात्मक समस्याएं आपकी सामाजिक प्रवृत्तियों (जैसे दोस्तों, रिश्तेदारोंआदि के साथ घूमना) में हस्तक्षेप किया है?

सभी समय १

ज्यादातर समय २

 कुछ समय ३

 थोड़ा समय ४

 कोई समय नहीं ५

निम्नलिखित में से प्रत्येक कथन आपके लिए कितना सत्य या असत्य है।

(प्रत्येक पंक्ति पर एक अंक का गोला बनाएं)

**Table 17:** RAND 36 - Item Health Survey (Hindi)

	निश्चित रूपसे सत्य	ज्यादातर सत्य	पता नहीं	ज्यादातर असत्य	निश्चित रूपसे असत्य
३३. मैं अन्य लोगो की तुलना में आसानी से बीमारहो जाता हूँ।					
३४. मैं किसी भी अन्य परिचित व्यक्ति की तरहस्वस्थ हूँ।					
३५. मुझे उम्मीद है कि मेरा स्वास्थ्य ज्यादा बिगड़ेगा।					
३६. मेरा स्वास्थ्य उत्तम है।					

Appendix F

RAND 36-Item Health Survey 1.0 Questionnaire Items (Gujarati Version)

Source: This scale was translated and validated in Gujarati by Jani et al. This scale was obtained by approaching the authors of the following article: https://ijshr.com/IJSHR_Vol.6_Issue.4_Oct2021/IJSHR08.pdf

Permission to reproduce this questionnaire has been obtained.

Questionnaire (Tables 18-23): 

**Table 18:** RAND 36 - Item Health Survey (Gujarati)

૧. સામાન્ય રીતે તમે કહો છો કે તમારું આરોગ્ય છે	
ઉત્તમ	૧
બહું સારું	૨
સારું	૩
બરાબર	૪
નબળું	૫
૨. એક વર્ષ પહેલાની તુલનામાં, હવે તમારા સ્વાસ્થ્યને સામાન્ય રીતે કેવી રીતે આંકન કરશો?	
એક વર્ષ પહેલા કરતા હવે ઘણું સારું	૧
એક વર્ષ પહેલા કરતા હવે થોડુંક સારું	૨
લગભગ સરખી	૩
એક વર્ષ પહેલા કરતા હવે કંઈક અંશે ખરાબ	૪
એક વર્ષ પહેલા કરતા હવે વધુ ખરાબ	૫

નીચેની મુજબની વસ્તુઓએ પ્રવૃત્તિઓ વિશે છે જે સામાન્ય દિવસ દરમિયાન કરી શકો છો. શું તમારું સ્વાસ્થ્ય હવે તમને આ પ્રવૃત્તિઓમાં માર્યાદિત કરે છે? જો એમ હોય તો કેટલું? (દરેક લીટી પર એક અંકને વર્તુળ કરો) 

**Table 19:** RAND 36 - Item Health Survey (Gujarati)

	हाँ, बहुत मर्यादित	हां, थोड़ा मर्यादित	ना, बिलकुलमरयित नहीं
૩. જોશીલી પ્રવૃત્તિઓ, જેમ કે દોડવું, ભારે પદાર્થોને ઉપાડવા, સખત રમતમાં ભાગ લેવો.	૧	૨	૩
૪. માધ્યમ પ્રવૃત્તિઓ, જેમ કે ટેબલ ખસેડવું, વેક્યૂમ ક્લીનર ને ધક્કો મારવો, દડો ફેકવો અથવા ગોલ્ફ રમવું.	૧	૨	૩
૫. કરિયાણું ઉચકવું અથવા લઇ જવું.	૧	૨	૩
૬. સીડીના ઘણા સમૂહ ચડવા.	૧	૨	૩
૭. સીડીનો એક સમૂહ ચડવો.	૧	૨	૩
૮. વળવું, નમવું અથવા ઘૂંટણિયે થવું.	૧	૨	૩
૯. એક માઈલ થી વધુ ચાલવું.	૧	૨	૩
૧૦. કેટલાક બ્લોક્સ ચાલવા.	૧	૨	૩
૧૧. એક બ્લોક ચાલવું.	૧	૨	૩
૧૨. નાહવું અથવા જાતે તૈયાર થવું.	૧	૨	૩

છેલ્લા ૪ અઠવાડિયા દરમિયાન, તમારે તમારા શારીરિક સ્વાસ્થ્યને પરિણામે તમારા કામ અથવા અન્ય નિયમિક દૈનિક પ્રવૃત્તિઓમાં નીચેની કોઈ સમસ્યા આવી છે? (દરેક લીટી પર એક અંકને વર્તુળ કરો)

**Table 20:** RAND 36 - Item Health Survey (Gujarati)

	હા	ના
૧૩. તમે કામ અથવા અન્ય પ્રવૃત્તિઓ માટે જેટલો સમય પસાર કર્યો છે તેમાં ઘટાડો કર્યો.	૧	૨
૧૪. તમારી ઈચ્છા કરતા ઓછા પરિપૂર્ણ	૧	૨
૧૫. કામના પ્રકાર અથવા અન્ય પ્રવૃત્તિઓમાં માર્યાદિત હતા	૧	૨
૧૬. કામ અથવા અન્ય પ્રવૃત્તિ કરવામાં મુશ્કેલી આવી હતી (ઉદાહરણ તરીકે, જેમાં વધુ પ્રયત્નો થયા)	૧	૨

છેલ્લા ૪ અઠવાડિયા દરમિયાન, તમને કોઈ ભાવનાત્મક સમસ્યાઓના પરિણામ રૂપે તમારા કાર્ય અથવા અન્ય નિયમિક દૈનિક પ્રવૃત્તિઓમાં નીચેની કોઈ સમસ્યા આવી છે? (જેમ કે હતાશા અથવા બેચેની) (દરેક લીટી પર એક અંકને વર્તુળ કરો)

**Table 21:** RAND 36 - Item Health Survey (Gujarati)

	હા	ના
૧૭. તમે કામ અથવા અન્ય પ્રવૃત્તિઓ માટે જેટલો સમય પસાર કર્યો છે તેમાં ઘટાડો કર્યો.	૧	૨
૧૮. તમારી ઈચ્છા કરતા ઓછા પરિપૂર્ણ.	૧	૨
૧૯. હંમેશની જેમ કાળજીપૂર્વક કામ અથવા અન્ય પ્રવૃત્તિઓ કરી નથી	૧	૨

૨૦. છેલ્લા ૪ અઠવાડિયા દરમિયાન, તમારા શારીરિક અઠવાડિયા દરમિયાન, તમારા શારીરિક સ્વાસ્થ્ય અથવા ભાવનાત્મક સમસ્યાઓએ કુટુંબ, મિત્રો, પાડોશીઓ અથવા જૂથો સાથેની તમારી સામાન્ય સામાજિક પ્રવૃત્તિઓમાં કેટલી હદે દખલ કરી છે? (એક નંબર ને વર્તુળ કરો)           

જરાય નહિ         ૧ 

સહેજ                ૨

સાધારણ           ૩  

થોડું ઘણું           ૪

અત્યન્ત             ૫

૨૧. છેલ્લા ૪ અઠવાડિયા દરમિયાન, તમને કેટલી શારીરિક પીડા હતી? (એક નંબર ને વર્તુળ કરો)           

કોઈ નહીં           ૧

ખૂબ હળવો        ૨

હળવો               ૩

સાધારણ           ૪

ગંભીર               ૫

ખૂબજ ગંભીર     ૬

૨૨. છેલ્લા ૪ અઠવાડિયા દરમિયાન, પીડા તમારા સામાન્ય કામમાં કેટલી દખલ કરે છે? (ઘરની બહાર અને ઘરના કામ બંને સહિત) (એક નંબર ને વર્તુળ કરો)

જરાય નહિ         ૧ 

થોડુંક                ૨

સાધારણ           ૩  

થોડું ઘણું           ૪

અત્યન્ત             ૫

આ પ્રશ્ન તમને કેવું લાગે છે અને છેલ્લા ૪ અઠવાડિયા દરમિયાન તમારી સાથે વસ્તુઓ કેવી રીતે રહી છે તે વિશે છે. દરેક પ્રશ્નો માટે, કૃપા કરીને એક જવાબ આપો જે તમારી અનુભૂતિની નજીક આવે છે. છેલ્લા ૪ અઠવાડિયા દરમિયાન કેટલો સમય છે...

(દરેક લીટી પાર એક અંક ને વર્તુળ કરો)

**Table 22:** RAND 36 - Item Health Survey (Gujarati)

	બધો સમય	મોટા ભાગના સમય	સારો એવો સમય	કેટલોક સમય	થોડો સમય	કોઈ સમય નહી
૨૩. શું તમે શક્તિથી ભરપુર અનુભવો છો?						
૨૪. તમે ખૂબ નર્વસ વ્યક્તિ છો?						
૨૫. શું તમને એટલું ઉદાસીન લાગ્યું છે કે કઈ પણ તમને ઉત્સાહિત કરી શકે નહીં?						
૨૬. શું તમે શાંતિ અનુભવી છે?						
૨૭. શું તમારી પાસે ખૂબ શક્તિ હતી?						
૨૮. શું તમે નિરાશ અને નિરુત્સાહનો અનુભવ કર્યો છે?						
૨૯. શું તમે થાકી ગયા છો?						
૩૦. શું તમે ખુશ વ્યક્તિ છો?						
૩૧. શું તમે થાક અનુભવો છો?						

૩૨. છેલ્લા ૪ અઠવાડિયા દરમિયાન , તમારી શારીરિક સ્વાસ્થ્ય અથવા ભાવનાત્મક સમસ્યાઓનો કેટલો સમય તમારી સામાજિક પ્રવૃત્તિઓમાં દખલ કરે છે (જેમ કે મિત્રો, સંભંધિઓ સાથે મુલાકાત કરવી વગેરે) (એક નંબર ને વર્તુળ કરો)

બધા સમયે         ૧ 

મોટે ભાગે          ૨

કેટલાક સમય     ૩

થોડો સમય        ૪

કોઈ સમય નથી ૫ 

તમારા માટે નીચે આપેલા દરેક નિવેદનોમાં કેટલું સાચું અથવા ખોટું છે?  (દરેક લીટી પાર એક અંક ને વર્તુળ કરો)

**Table 23:** RAND 36 - Item Health Survey (Gujarati)

	ચોક્કસપણે સાચું	મોટા ભાગે સાચું	ખબર નથી	મોટે ભાગે ખોટું	ચોક્કસ ખોટું
૩૩. હું અન્ય લોકો કરતા થોડો સહેલાઈથી બીમાર પડું છું.					
૩૪. હું જે કોઈને જાણું છું તેટલો સ્વસ્થ છું.					
૩૫. હું અપેક્ષા કરું છું કે મારી તબિયત વધુ ખરાબ થાય.					
૩૬. મારું સ્વાસ્થ્ય ઉત્તમ છે.					
